# Incidence and influencing factors of fertility concerns in breast cancer in young women: a systematic review and meta-analysis

**DOI:** 10.3389/fonc.2023.1273529

**Published:** 2023-12-19

**Authors:** Lijuan Chen, Jiali Shen, Hongzhan Jiang, Huihui Lin, Jiaxi He, Siyue Fan, Liping Yang, Doudou Yu, Rongliang Qiu, Ende Lin

**Affiliations:** ^1^ Department of General Surgery, Zhongshan Hospital of Xiamen University, School of Medicine, Xiamen, China; ^2^ School of Nursing, Fujian University of Traditional Chinese Medicine, Fuzhou, China; ^3^ School of Medicine, Xiamen University, Xiamen, China; ^4^ The Third Clinical Medical College of Fujian Medical University, Fuzhou, China

**Keywords:** breast cancer, fertility concerns, women, meta-analysis, systematic review

## Abstract

**Objective:**

This systematic review and meta-analysis aimed to evaluate the prevalence and influencing factors of fertility concerns in breast cancer in young women.

**Methods:**

A literature search on PubMed, Embase, Web of Science, and Cochrane Library databases was conducted up to February 2023 and was analyzed (Revman 5.4 software) in this study. The papers were chosen based on inclusion standards, and two researchers independently extracted the data. The included studies’ quality was evaluated using criteria set out by the Agency for Healthcare Research and Quality. To identify significant variations among the risk factors, odds ratios (ORs) and the corresponding 95% confidence intervals (CIs) were utilized.

**Results:**

A total of 7 studies that included 1579 breast cancer in young women were enrolled in the study. The results showed that for breast cancer in young women, the incidence of fertility concerns 53%(95%*CI* [0.45,0.58]). The results showed that education (2.65, 95% *CI* 1.65–5.63), full-time work (0.12, 95% *CI* 1.03–1.93), fertility intentions (7.84, 95% *CI* 1.50–37.4), depression level (1.25, 95% *CI* 1.03–1.5), and endocrine therapy (1.32, 95% *CI* 1.08–1.62) were risk factors for fertility concerns in young women with BC. Having a partner (0.41, 95% *CI* 0.33–0.5), ≥1 child (0.3, 95% *CI* 0.22–0.4) were identified as protective factors against fertility concerns in young women with BC.

**Conclusions:**

The incidence of fertility concerns in breast cancer in young women is at a moderately high level. We should pay more attention to the risk factors of fertility concerns to help breast cancer in young women cope with their fertility concerns and promote their psychological well-being.

## 1 Introduction

Breast cancer (BC) has the highest frequency of occurrence among global malignancies and stands as the leading cause of death in young women aged ≤40 years (1, 2). BC rates continue to rise in younger women, with an estimated 12,000 cases diagnosed annually in the United States (3). In other advanced nations, the prevalence of BC, specifically among women below the age of 40 years, is estimated to be 5–7% of all BC cases (4). BC therapies have both immediate and enduring detrimental effects on fertility, primarily stemming from the harm inflicted upon ovarian function. This harm results in conditions such as amenorrhea, premature menopause, and diminished fecundity. Therefore, young patients with BC who desire to pursue conception may face challenges because these consequences can contribute to fertility concerns (5). Consequently, patients worry about disease progression, lifespan expectations, communication with partners, emotional adaptation to potential infertility, and fertility well-being (6).

In recent years, increasing attention has been paid to oncofertility care in young adult patients with cancer (7). According to previous studies, nearly half of young individuals seeking medical care for cancer treatment also experience fertility issues (8). Additionally, >80% of young patients with cancer express a desire for spontaneous conception (9). Fertility concerns may impact patients more than the cancer itself, because they last longer and significantly reduce the quality of life of young women with BC (10). In accordance with all guidelines, patients diagnosed with any malignancy and stage at a reproductive age must receive adequate counseling on the dangers of gonadotoxicity caused by anticancer treatment at the time of diagnosis (11, 12). The management of oncofertility treatment in young women with breast cancer requires unique considerations (13). Fertility preservation and the desire for pregnancy should be pivotal in addressing fertility concerns in young women with BC (14). Despite being a major concern for patients, the adoption of fertility preservation options has been limited (15).

Therefore, determining the factors that influence fertility concerns in young women with BC is important to alleviate such concerns. Established risk factors for BC include aging, education, depression, and fertility. However, recent studies have introduced some controversies. For instance, a study by Villarreal Garza et al. reported that age is an influential factor in fertility concerns among patients with BC (9). In contrast, research conducted by Gorman et al. showed that fertility concerns among patients receiving chemotherapy are unrelated to their age (16). The present study aimed to investigate the frequency and factors contributing to fertility concerns in patients with BC through a systematic review and meta-analysis. By analyzing existing literature, the goal was to identify factors influencing fertility concerns and offer evidence-based recommendations to clinicians regarding fertility preservation and posttreatment pregnancies.

## 2 Materials and methods

This systematic review was conducted according to the recommendations of the Preferred Reporting Items for Systematic Reviews and Meta-analyses (PRISMA) (17). The study protocol has been registered in PROSPERO (ID: CRD42023412503).

### 2.1 Search strategy

Databases such as PubMed, Embase, Cochrane Library, and Web of Science were used for literature searches up to February 2023, using the following keywords: (“breast neoplasm*” OR “breast tumor*” OR “breast cancer*” OR “breast carcinoma*” OR “mammary cancer*” OR “mammary neoplasm*” OR “mammary carcinoma*”) AND (“fertility concerns” OR “fertility-related concerns” OR “reproductive concerns” OR “childbearing concerns “ OR “pregnancy concerns”).

### 2.2 Selection of studies

To be included in this systematic review and meta-analysis, eligible studies had to meet the following inclusion criteria: (i) the study was conducted on female patients aged 18–40 years; (ii) patients were diagnosed with BC through pathological examination; (iii) a scale assessed the level of fertility concerns in patients; (iv) the type of study was observational; and (v) the studies were in the English language.

The exclusion criteria were: (i) conference abstracts, (ii) literature for which full text was not available or duplicate publications, (iii) literature from which data could not be extracted, and (iv) literature with a quality evaluation of <3 points.

### 2.3 Data extraction and quality assessment

Two researchers (Jiali Shen and HongZhan Jiang) independently screened the literature according to the inclusion standards. They excluded the literature that was irrelevant to the topic or appeared repeatedly. Subsequently, they read the remaining literature in full to determine the final selection for this study, and finally extracted and cross-checked the data. In case of disagreement or other issues, a third party (Huihui Lin) made the final decision. The first author, title, publication date, sample size, measurement tools, and patient age were extracted from all included studies.

The evaluation of literature quality was independently conducted by two researchers using the evaluation criteria recommended by the Agency for Healthcare Research and Quality (18). The evaluation comprised 11 items, which were answered with “Yes,” “No,” or “Unclear.” Each answer of “Yes” was scored as one point, while the opposite was scored as zero points, out of a total of 11 points. A score of ≥8 indicated high quality, 4–7 denoted medium quality, and ≤3 indicated low quality. In cases of disagreement, an agreement was reached through discussion.

### 2.4 Data analysis

Statistical analyses were performed using RevMan 5.4 software (https://revman.cochrane.org). The odds ratio (OR) or relative risk (RR) values (95% confidence interval [CI]) for factors influencing fertility concerns in young female patients with cancer were extracted as effect sizes. Heterogeneity was assessed using the Q test. If *P >*0.1 and *I* ^2^ <50%, it indicated no significant heterogeneity among the studies, and a fixed-effect model was selected; conversely, if *P <*0.05, it indicated a significant difference, and a random-effect model was selected. To assess the reliability of the meta-analysis, random- and fixed-effect models were analyzed separately, and the robustness of the meta-analysis results was calculated.

## 3 Results

### 3.1 Study selection

**Figure 1:**
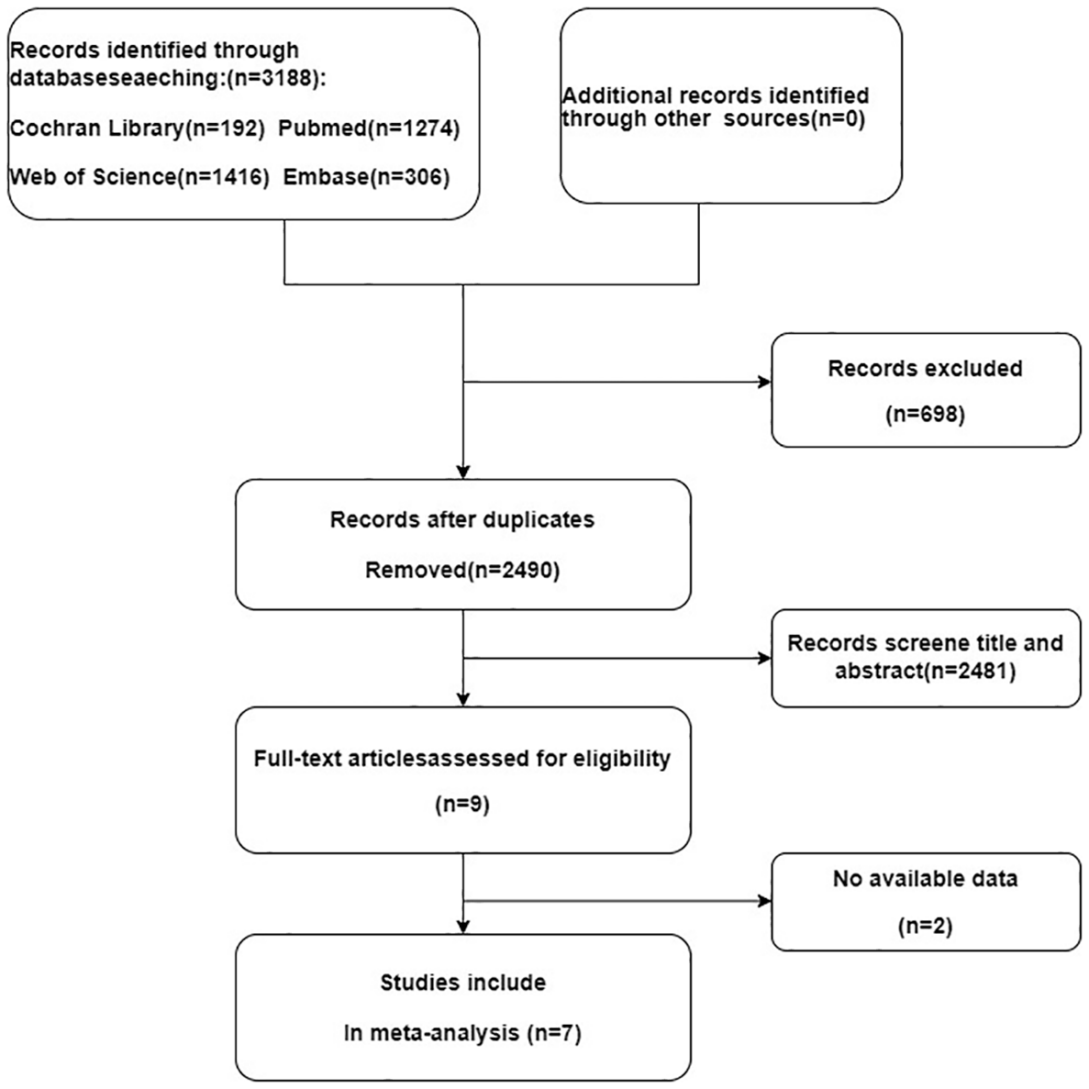
Study flow diagram.

Following an initial literature search across PubMed, Embase, Cochrane Library, and Web of Science databases, 2041 articles were identified. After excluding duplicates and irrelevant studies (Endnote X 9.1), 1343 potentially relevant articles remained. Among these, 1334articles were excluded after reviewing titles or abstracts. After thoroughly reading the full texts of the remaining nine articles, two studies were excluded due to the unavailability of data. Finally, seven studies were included in this meta-analysis (**Figure 1**).

### 3.2 Characteristics of the included studies

Seven studies included a total of 1579 patients, including five cross-sectional studies (9, 16, 19–21) and two prospective cohort studies (22, 23). Five studies (9, 16, 19, 22, 23) were of high quality, and two (20, 21) were of moderate quality, resulting in an overall moderate to high quality. The basic characteristics and quality evaluation scores of the included studies are listed in **Table 1**.

**Table 1:** The characteristics of the included studies.

Author	Year	Country	Instrument	Sample size	Aged(Years)	Type of Study	Prevalence	Influence factor	Quality assessment
Villarreal Garza C et al. (12)	2017	Mexico	FIS	134	34.6	Cross-sectional	44%	CDFGHIJ	8
Gorman JR et al. (13)	2010	USA	RCS	131	36.7	Cross-sectional	64%	AFG	8
Ba´rtolo A et al. (16)	2019	Portuguesa	RACA	104	36.1 ± 3.03	Cross-sectional	36%	K	6
LjungmanL et al. (17)	2018	Sweden	RACA	181	34.6 ± 4.1	Cross-sectional	56%	BFIJ	9
Ruggeria M et al. (18)	2014	Switzerland Italy	RACA	297	18-40	Cohort study	58%	ABCDEGHIJL	8
Ruddy KJ et al. (19)	2014	USA	FIS	620	17-40	Cohort study	51%	ABCDEGHIJKL	8
Jiajia Qiu et al. (20)	2022	China	RACA	112	21-40	Cross-sectional	–	ABG	6

A, age; B, education; C, With partner; D, economic; E, genetic; F, desire to have children; G, >1 child; H, surgery; I, chemotherapy; J, endocrine therapy; K, depression; L, full-time job FIS, Fertility Issues and Outcomes Scale; RCAC, Reproductive Concerns After Cancer Scale; RCS, reproductive concerns scale.

"–", No data available.

### 3.3 Prevalence of fertility concerns among breast cancer patients

The prevalence of fertility concerns in young women with BC ranged from 36% to 64%, and heterogeneity was observed after combination treatment (*I* ^2 ^= 100%, *P <*0.00001). Therefore, using a random-effect model, the prevalence of fertility concerns in young women with BC after combination therapy was 53% (95% *CI* 0.45–0.58).

### 3.4 Factors affecting fertility concerns

Two studies revealed depression, four reported ≥1 child, three reported endocrine therapy, and four reported genetic factors (cancer in the immediate family) as influencing factors of fertility concerns in young women with BC, which showed less heterogeneity (*I* ^2^ ≤50%, *P >*0.1). Therefore, a fixed-effect model was used. Four studies revealed age, four reported education, three reported having a partner, three reported economic level, three reported fertility intention, three reported surgery, four reported chemotherapy, and two reported full-time work as factors influencing fertility concerns in young women with BC, and these were found to be heterogeneous (*I* ^2^ ≥50%, *P <*0.1). Thus, a random-effect model was used.

**Figure 2:**
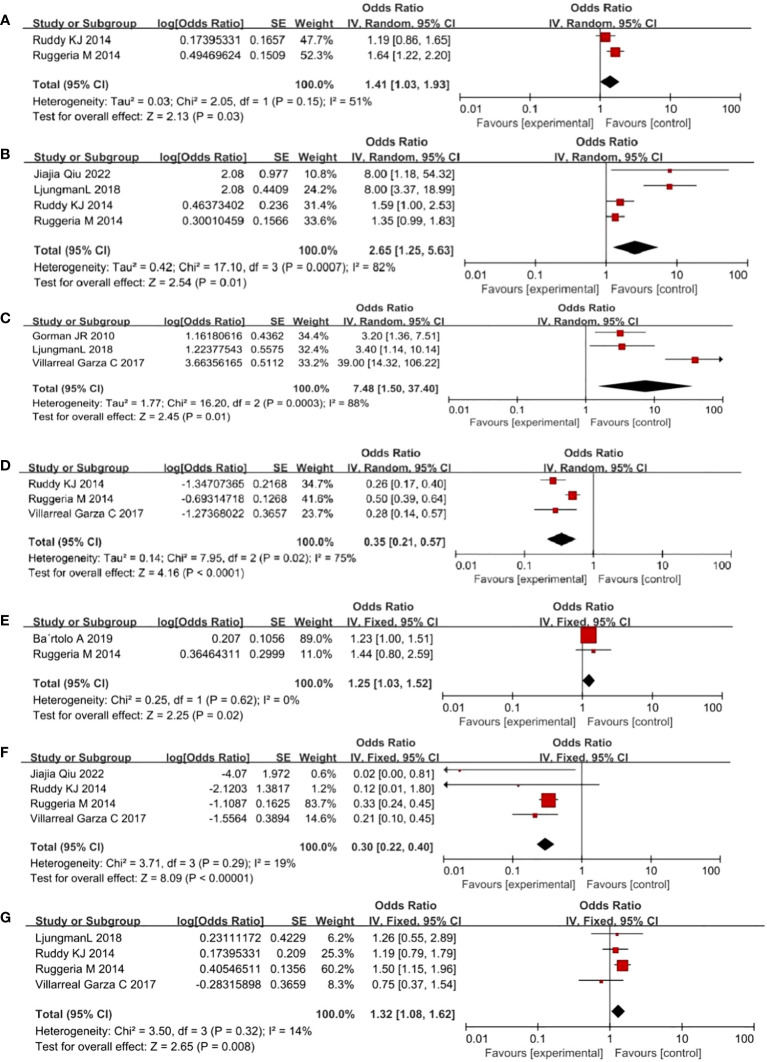
Forest plot of risk factors in breast cancer in young women. (**(A)** full-time work **(B)** education **(C)** desire to have children **(D)** with partner **(E)** depression **(F)** ≥ 1 child **(G)** endocrine therapy. Horizontal lines: 95% confidence intervals for study results, Square boxes: Effect quantity for a single study, Diamonds: Merged results, Vertical lines: Invalid line, determining whether the difference in results is statistically significant or not.).

The results showed that education (2.65, 95% *CI* 1.65–5.63), full-time work (0.12, 95% *CI* 1.03–1.93), fertility intentions (7.84, 95% *CI* 1.50–37.4), depression level (1.25, 95% *CI* 1.03–1.5), and endocrine therapy (1.32, 95% *CI* 1.08–1.62) were risk factors for fertility concerns in young women with BC. Having a partner (0.41, 95% *CI* 0.33–0.5), ≥1 child (0.3, 95% *CI* 0.22–0.4) were identified as protective factors against fertility concerns in young women with BC (**Table 2**; **Figure 2**).

**Table 2:** Meta-analysis of risk factors of fertility concerns in breast cancer patients.

Influencing factors	Combination studies	*Heterogeneity of* *study design*		Analysis model	OR (95%*CI*)	*P*_2_	Egger’s test
		*I* ^2^	*P*_1_				
Full-time work	2	51%	0.15	Random	1.41[1.03,1.93]	**0.03**	NA
Age	4	97%	<0.00001	Random	0.63[0.14,2.85]	0.54	0.761
Education	4	82%	0.0007	Random	2.65[1.65,5.63]	**0.01**	0.075
Economic	3	55%	0.11	Random	0.89[0.63,1.26]	0.51	0.536
Fertility intentions	3	88%	0.0003	Random	7.84[1.50,37.40]	**0.01**	0.815
With partner	3	75%	0.02	Random	0.41[0.33,0.50]	**<0.00001**	0.374
Depression	2	0%	0.62	Fixed	1.25[1.03,1.52]	**0.02**	NA
≥1 child	4	19%	0.29	Fixed	0.3[0.22,0.40]	**<0.00001**	0.273
Surgery	3	71%	0.03	Random	0.72[0.47,1.11]	0.14	0.859
Chemotherapy	4	55%	0.08	Random	1.38[0.96,2.00]	0.08	0.433
Endocrine therapy	4	14%	0.32	Fixed	1.32[1.08,1.62]	**0.008**	0.225
Genetic	2	0%	0.92	Fixed	1.07[0.71,1.60]	0.74	NA

NA: Insufficient number for Egger’s test.The bold values: Nominal p-value <0.05.

### 3.5 Sensitivity analysis and publication bias

The sensitivity analysis of the seven studies was performed by excluding each study one by one, and the results did not change significantly, suggesting good stability of the results. Egger’s test was conducted to evaluate potential publication bias. The results revealed that most risk factors did not have publication bias (*P >*0.05; **Table 2**).

## 4 Discussion

The seven studies included in this systematic review and meta-analysis specified the inclusion and exclusion criteria of the study patients, assessment methods, and correct use of statistical methods. The quality of the literature met the requirements with high reliability. The prevalence of fertility concerns among young patients with BC in this study was 53%, which is relatively high. It is lower than the results of the high-concern group by Gorman JR et al. in the United States at 56% (16); however, it is higher than the results reported by Bartolo A et al., where 35.6% of this sample presented moderate-to-high overall reproductive concerns in Portugal (20). This difference may be attributed to variations in countries, cultures, and ideologies. Nevertheless, our study shows that a significant proportion of patients with BC are already affected by fertility concerns or will be affected shortly. Therefore, more attention should be paid to helping these patients cope with their fertility concerns and promoting their psychological well-being.

Education was identified as a risk factor for the development of fertility concerns in young women with BC, likely related to the increased informational needs of more literate patients about the disease and fertility. Studies have indicated that highly educated BC patients require more information (24). Patients are more prone to experiencing emotional issues, such as despair and anxiety, when their information needs are not being satisfied. A foreign survey (25) revealed that only 30% of patients with cancer had received health guidance on fertility protection, and merely 23.4% of patients with BC received fertility counseling during treatment (26). In the United States and the United Kingdom, 30–60% of cancer survivors of reproductive age reported having no information on cancer fertility protection (27). Healthcare professionals should prioritize enhancing fertility-related information and education after BC diagnosis to provide patients with high-quality, credible, and evidence-based information about fertility through various channels, including the internet and social media. Thus, the fertility knowledge needs of patients with BC can be met.

Full-Time work increases the risk of reproductive issues in young women with BC, potentially due to concerns that their jobs might be negatively impacted by disease treatment (28). Patients whose work is affected may face a greater financial burden, including the cost of treatment, costs related to the risk of future pregnancy and potential health problems of their children. This situation can lead to difficulties in fertility decision-making and heightened levels of fertility concerns (29). Providing adequate information support, especially for patients working full-time, is important. Fertility education has a positive effect on improving disease-related knowledge and anxiety. Stark et al. (30) and Su et al. (31) developed an internet-based survivor reproductive healthcare program that provided online reproductive health and fertility education; it reduced the level of fertility concerns among BC survivors. Fertility counseling and educational intervention services are required to lessen the lack of knowledge that causes concerns about fertility.

Fertility intention was identified as a risk factor for fertility concerns, representing the subjective expectation of having children. This expectation involves considerations such as the number, sex, timing, spacing, and quality of children. Additionally, this expectation stems from a person’s basic needs and is influenced by various circumstances (32). When their behavior does not meet their psychological expectations for various reasons, they experience different degrees of apprehension. Patients with strong fertility intentions are more concerned about the impact of tumors and related treatments on their reproductive function, and excessive attention to this aspect is likely to cause heightened worry. According to some studies, implementing fertility preservation can alleviate patients’ concerns about fertility (33). Several nations have developed guidelines for fertility preservation in oncology that suggest providing patients fertility preservation counseling before treatment (11, 12). The guidelines and practicing physicians suggest the use of embryo cryopreservation, cryopreservation of unfertilized oocytes, ovarian transposition and suppression, and ovarian tissue cryopreservation and transplantation for fertility preservation (34). The implementation of fertility preservation still requires the joint efforts of the government and all medical personnel.

In this study, depression was found to be a risk factor for fertility concerns. The news of a tumor diagnosis is extremely upsetting for the patient and can result in decreased quality of life, fewer social opportunities, and increased financial burden—all of which can quickly lead to depression. Fertility concerns remain a contributor to depression risk, with each additional contributor increasing the likelihood of depression by 2.423 times (35). BC may impair the integrity of the secondary sexual characteristics in female patients, which may lead to long-term feelings of self-blame and inferiority, making it more likely to cause depression (36). Therefore, medical personnel should pay attention to patients’ psychological states to identify and provide timely relief. According to practicing physicians’ recommendations, positive stress-reduction therapy reduces depression levels. For example, encouraging an increase in physical activity (37), contemplation (38), and mindfulness-based stress reduction (39) can improve mental health and reduce depression levels.

Studies have shown that having ≥1 child and having a partner are protective factors against fertility concerns in patients with BC. As the basic unit of society, a sound family typically includes children; therefore, patients who already have children do not face the demands and pressure from their husbands and other family members to have more children (40). Children can also act as caregivers, sharing the emotional and psychological stresses that tumors bring to patients. Patients with partners tend to have more stable and intimate relationships. Moreover, good family intimacy can enhance the willingness and efficacy of self-expression between patients and their family members. Patients can actively and flexibly utilize the available resources around them, obtaining emotional and material support from their spouses, family members, and friends; the more tolerance and care patients feel, the better it helps them readjust to stressful events. Additionally, the patient’s self-expression process helps family members or friends identify the patient’s concerns and provide relief. This, in turn, stimulates the patient to express positive emotions, build positive cognition, and alleviate concerns due to fertility problems (41).

### 4.1 Study limits

This systematic review and meta-analysis had several limitations. First, we included only English-language literature from the four databases, which may have resulted in insufficient retrieval. Second, some risk factor indicators in this meta-analysis were not combined effectively because of the limited amount of available literature, which may have affected the results. Third, because the research methods, study populations, and observational periods varied among the included studies, the findings were dispersed and heterogeneous. Therefore, prospective cohort studies with multicenter approaches and larger sample sizes are required to increase the effect size and enrich the results.

## 5 Conclusion

Through this systematic review and meta-analysis, we examined the prevalence of and factors contributing to fertility concerns among young women with BC. The results revealed a high prevalence of concern about fertility in this population. Education, full-time work, fertility intentions, depression, and endocrine therapy were identified as risk factors for fertility concerns in young women with BC. Having partners and ≥1 child were protective factors against fertility concerns in young patients with BC. The results of the present study can be used as a basis for better planning to address fertility concerns and offer evidence-based recommendations for clinicians regarding fertility preservation and post-treatment pregnancies.

## Author contributions

JS: Writing – original draft, Writing – review & editing. HJ: Data curation, Supervision, Validation, Writing – review & editing. HL: Supervision, Writing – review & editing. EL: Supervision, Validation, Writing – review & editing. JH: Conceptualization, Data curation, Supervision, Writing – review & editing. RQ: Conceptualization, Methodology, Supervision, Writing – review & editing. SF: Supervision, Validation, Writing – review & editing. DY: Data curation, Investigation, Supervision, Visualization, Writing – review & editing. LY: Data curation, Methodology, Supervision, Writing – review & editing. LC: Writing – review & editing.
